# ETS1 is associated with cisplatin resistance through IKKα/NF-κB pathway in cell line MDA-MB-231

**DOI:** 10.1186/s12935-018-0581-4

**Published:** 2018-06-19

**Authors:** Yuzhu Zhang, Jingjing Wu, Meina Ye, Bing Wang, Jiayu Sheng, Bailing Shi, Hongfeng Chen

**Affiliations:** 1 Department of Breast, Longhua Hospital Affiliated to Shanghai University of TCM, No.725, Wanping South Road, Xuhui District, Shanghai, 200032 China; 2 Department of Breast Surgery, Shanghai Yueyang Hospital of Integrated Traditional Chinese and Western Medicine, Shanghai, China; 3 Department of Thyroid and Breast Surgery, The Third Affiliated Hospital of Zhejiang University of Traditional Chinese Medicine, Hangzhou, China; 4 Department of Mammary Disease, Guangdong Provincial Hospital of Chinese Medicine, Guangzhou, Guangdong People’s Republic of China

**Keywords:** DDP-resistance, ETS1, NF-κB, IKKα, ShRNA, Survival

## Abstract

**Background:**

Platinum-based drugs are used extensively in neoadjuvant chemotherapy for triple-negative breast cancer (TNBC), but their use can be limited by resistance. In this study, we established cisplatin (DDP) resistant TNBC cells to investigate the potential relationship among ETS1, IKKα/NF-κB and resistance.

**Methods:**

The sensitivity was evaluated by MTT, apoptosis analysis. The intracellular DDP concentration difference was tested by inductively coupled plasma mass spectrometry (ICP-MS) method. Molecular pathological mechanism of DDP resistance was explored by microarray analysis and PPI network analysis. The ETS1, NF-κB signaling change were assessed by western blot and q-PCR in vitro and vivo. The existing binds between ETS1 and the core IKKα promoter were found by luciferase assay and chromatin immunoprecipitation technique (ChIP).

**Results:**

MDA-MB-231/DDP (231/DDP) cell had a higher IC_50_ value of cisplatin, lower intracellular DDP concentration, and lower apoptosis ratio than MDA-MB-231 (231/wt) cell line treated with DDP. Increased ABC transporters were induced by the activation of NF-κB pathway in 231/DDP cells. ETS1, RPL6, RBBP8, BIRC2, PIK3A and RARS were six important genes for DDP-resistance based on PPI network and expression validation. Protein expression of ETS1 and IKKα were significantly up-regulated in 231/DDP cells. However, inhibition of ETS1 expression enhances chemo-sensitivity to DDP and reversed the activation of NF-κB pathway in 231/DDP cells and subcutaneous transplantation tumor in vivo. Moreover, there is existing binds between ETS1 and the core IKKα promoter though luciferase assay and ChIP.

**Conclusion:**

This study enables us to understand the functions of ETS1 in TNBC chemotherapy and suggests that ETS1 could be used as a novel marker of poor response to DDP and a potential therapeutic target for TNBC chemotherapy.

## Background

Triple-negative breast cancer (TNBC) is an aggressive histological subtype, which is known as a breast cancer that lacks expression of ER, PR and HER2 [1] and accounts for 15% of all breast cancer types. Currently, chemotherapy is the main treatment for TNBC [2], such as anthracycline and paclitaxel. However, due to p53 mutation in TNBC anthracycline-related drug resistance has been reported [3]. Cisplatin has an effective role in breast cancer, especially BRCA1-associated cancers, which are normally triple-negative type [4]. A previous study found that the response to DDP-based drugs in breast cancer with *BRCA1*^*C61G*^ mutation is poorer than that with homozygous *BRCA1* mutation [5]. The secondary mutation contributes to the restoration of reading frame of BRCA1 protein [6]. Therefore, it is supposed that revertant mutation might be a source of resistance to DDP in TNBC.

In our study, the TNBC cell line 231/wt and DDP-resistant cell line 231/DDP were used. Differences were compared between chemo-sensitivity to different drug agents, intracellular DDP accumulations and apoptosis. We found: all results show that the cells line 231/DDP have DDP-resistance character; then, increased ABC transporters are induced by the activation of NF-κB pathway in 231/DDP cells.; furthermore, ETS1, RPL6, RBBP8, BIRC2, PIK3A and RARS are six important genes for DDP-resistance based on microarray analysis, PPI network and expression validation. However, it had been reported enforced over-expression of ETS1 induced IKKα mRNA and protein expression as well as IKKα promoter activity [7]. Our results suggested that the protein expression of ETS1 and IKKα are significantly up-regulated in 231/DDP cells. In addition, inhibition of ETS1 expression enhanced chemo-sensitivity to DDP and reversed the activation of NF-κB pathway in 231/DDP cells. Besides, stable knocking-down ETS1 increased the efficacy of DDP in mouse xenograft models.

## Methods and materials

### Cell lines and cell culture

The TNBC cell line 231/wt was bought from the cell resource center of Shanghai Institutes for Biological Sciences, Chinese Academy of Sciences. The DDP-resistant human TNBC cell line 231/DDP was obtained by stimulating 231/wt cell lines with different DDP concentrations, as described in our previous work [8]. These cell lines were recovered in the medium without DDP, then was cultured in the medium with DDP (1.5 μg/mL) on the next day in the atmosphere of 5% CO_2_ at 37 °C.

### Detection of intracellular DDP concentration using ICP-MS method

The 1 μg/mL DDP was added in both of the 2 cell lines at the logarithmic phase, when the cell inhibitory concentration (IC_50_) value was “0”. After 48 h pretreatment, the supernatant was abandoned, and the cells were washed in PBS for 3 times, and then collected by cell scraper. Thereafter, cells were suspended in lysis buffer containing concentrated nitric acid, and incubated at 60 °C for 20 min. Then, intracellular DDP concentration was measured. The splitting cells were transferred into a 1.8 mL EP centrifugal tube, and then put these into liquid nitrogen and water bath for 1 min, respectively. After repeating for 3 times, the DDP concentration was measured based on the inductively coupled plasma mass spectrometry (ICP-MS) method [9].

### Cell viability

MTT assay was used to measure the drug resistance of DDP-resistant cell line. In brief, cells were inoculated in the 96-well plate, 5000 cells per well. After 24 h incubation, if all cells were attached, which could be separately treated with four drugs as DDP, doxorubicin (DOX), paclitaxel and cyclophosphamide (CTX). The specific operation steps is that DDP was dissolved in amide,*n*,*n*-dimethyl-formicaci (DMF) (2 mg/mL, 6.67 mM) then diluted by phosphate buffer solution (PBS). DOX was dissolved in dimethyl sulfoxide (DMSO) (2 mg/mL, 3.45 mM) then diluted by PBS. CTX was dissolved in DMSO (2 mg/mL, 7.17 mM) then diluted by PBS. Paclitaxel was dissolved in DMSO (2 mg/mL, 2.34 mM) then diluted by PBS. After 48 h treatment, the cells were incubated in MTT for 4 h, and then DMSO was added and shocked for 10 min. OD value of the survival cells were determined under 490 nm wave, with the reference under 630 nm. On this basis [10], the IC_50_ value in each group was calculated.

### Flow cytometric analysis of apoptosis

Extent of apoptosis was measured by Annexin V-FITC apoptosis detection kit (Invitrogen Corporation, California, USA) according to supplier’s instruction. Briefly, cells were treated with 1 μg/mL DDP for 48 h, collected and stained with Annexin V-FITC, then analyzed by using FACScan flow cytometer (Becton–Dickinson, Newjersey, USA).

### Microarray analysis and PPI network analysis

There were six samples in the following GeneChip probe array analysis: DDP-resistant (231/DDP, n = 3) and non-resistant breast cancer cell lines (231/wt, n = 3). First, total RNAs in each sample were extracted by the TRIzol method (Invitrogen), and then examined by the Agilent 2100 Bioanalyzer (Agilent Technologies, Palo Alto, CA). Next, the RNAs were amplified and labeled using the GeneChip WT PLUS reagent kit (Affymetrix, Santa Clara, CA, USA) according to the manufacturer’s instruction. Afterwards, the labeled RNAs were hybridized to Affymetrix GeneChip Human Transcriptome Array 2.0 in hybridization oven (Affymetrix model 640) for 16 h at 45 °C [11, 12].

### Quantitative real-time reverse transcription-PCR

As aforementioned, total RNA was extracted by the TRIzol method (Invitrogen), and was then reversely transcribed into cDNA according to the manufacturer’s instruction of the TaqMan reverse transcription kit (Applied Biosystems) (Logan et al. 2006). Following, the quantitative real-time reverse transcription-PCR (qRT-PCR) was performed to calculate gene expressions, with GAPDH as the internal reference. Primer sequences of the genes are listed in Table 1. Rotor-Gene6 software was used to calculate the Ct value, and gene expressions were calculated by 2^−ΔΔCt^ method [13].

**Table 1:** Primer sequences of genes

Gene	Primer (5′ to 3′)	Annealing temperature (°C)
ETS1	F: TTACTCAGCGCCTCGTCCT	59.9
	R: GATCCCCAGTCGTTGCTGTT	
RPL6	F: TCAGAGGAATTGGCAGGTA	61.4
	R: AGGCACATCTTCAGTAGGA	
BIRC3	F: TTGTGATGGTGGCTTGAG	61.2
	R: AGTGGTATCTGAAGTTGACA	
RBBP8	F: TGAAGAAGCAAGAGCAGAA	58.1
	R: TGGAATGTAGCGGAATCG	
RARS	F: GAAGCGAGCATATCAGTGT	58.3
	R: AGCCAGGTCAGATGTATCA	
PIK3A	F: CGAGGTTTTGCTGTTCGGTG	62.4
	R: CAGGCCAAACCTCTGGCTAA	

### Western blot

Total proteins were extracted and protein concentrations were evaluated using Bradford assay (Pierce Biotechnology Inc., Rock-ford, USA). Western blotting assay was performed as described previously [3]. The antibodies against ETS1(ab26096), MRP2(ab3373), BRCP(ab3380), P-gp(ab103477), P38(ab31828), p-P38(ab45381), IKKα(ab32041), IKKβ(ab32135), and NF-κB (ab16502) were purchased from Abcam.

### shRNA transfection

PHY-310-GFP plasmid expressing human ETS1 was provided by Hanyi Biotechnology Shanghai. For the knockdown experiment, the ETS1 shRNA (5′-CGCUAUACCUCGGAUUACU-3′) was cloned into PHY-31-GFP vector. The 231/DDP cells were subcultured in a 6-well plate for 24 h, with a concentration of 70–80%. 231/DDP was transfected stably PHY-310-GFP-shETS1 by using Lipofectamine™ 2000 Reagent (Invitrogen, USA), followed by selection in 2-μg/mL puromycin. Stable ETS1 knockdown cells were used in the subsequent study.

### Immunofluorescence assay

Cells were placed on a glass slide at the density of 1.0 × 10^5^ cells/mL. Cultured for 48 h, the cells washed for with PBS and then fixed with 4% paraformaldehyde for 20 min at 4 °C. Cells were permeabilized using 0.3% Triton X-100/PBS for 20 min and washed with PBS. The cells were then incubated for 30 min in the 0.5% BSA. Diluted primary antibody (1:50) was applied to each coverslip and incubate overnight at 4 °C. After washing in PBS, the cells were incubated with rabt antibody at a 1:200 dilution for 1 h at room temperature in the dark and followed by DAPI nuclear staining (1 μg/mL) for 5 min. After washing, the images were taken using a Zeiss Laser Scanning Confocal Microscope (LSM7 DUO).

### IKKα promoter and luciferase activity assays

To construct the IKKα promoter, we amplified the promoter region from − 217 to − 2216 from transcription start site by PCR using hot-start DNA polymerase (New England Biolabs, Ipswich, Massachusetts, USA). A 3-kb fragment containing *Kpn*I and *Xho*I sites was then ligated into the PCR2 vector by using a Plasmid minni Kit I (200) (OMEGA). The promoter fragment was excised from IKKαby digesting it with *Kpn*I and *Xho*I and then clone in PGL3-Basic vector (IKKα-NC) and IKKα-luciferase vector (IKKα-OE) to produce promoter luciferase report.

Sub-confluent cells cultured in 6-well plates were transiently co-transfected with luciferase reporter and internal control plasmids, using the DNAfectin Transfection Reagent according to the manufacture’s protocol. Cells were lysed in passive lysis buffer, and luciferase assay was performed using dual-luciferase reporter assay kit in 48 h post transfection. Renilla luciferase activity was used as control for transfection was performed in triplicate.

### Chromatin immunoprecipitation (ChIP)

ChIP was performed using ChIP assay kit (Cell Signaling Technology, CST, USA) according to the manufacture`s instruction. The main step is that cells in a 10-cm culture plate were crosslinked for 10 min by 1% formaldehyde. Crosslinking was neutralized by 0.2 M glycine. Cells were collected and suspended in lysis buffer (25 mM Tris–HCl, 150 mM NaCl, 1 mM EDTA, 1% SDS and 5% glycerol). Genomic fragments were sonicated to a proper length. Protein–DNA complexes were precipitated with ETS1 antibody or immunoglobulin G overnight at 4 °C. The complexes were purified by protein A/G magnetic beads and the crosslinks were reversed at 68 °C. Quantitative PCR was performed, after DNA fragments were purified. Primer sequences used in ChIP-qPCR are as follows: IKKa 5′-GTGGTTCCGTTCAGCCCT-3′ (Forward), 5′-TGCTCGCGCGTCTTTG-3′ (Reverse); U2 5′-ATCGCTTCTCGGCCTTTTGG-3′ (Forward), 5′-AGGTCGATGCGTGGAGTGGA-3′ (Reverse).

### Mouse xenograft studies

Four-week old female BALB/C nude mice, weight of 15–16 g, were obtained from Shanghai SLAC Laboratory Animal Company (Shanghai China). The mice were housed specific pathogen-free conditions at 22 ± 2 °C, at 70% relative humidity and under a 12-h light/dark cycle. Six-week old female nude mice, approximately 20 g, were transfected 231/DDP cells (sh-ETS1 and empty vector) were harvested at a concentration of 1.0 × 10^7^ cells/mL and suspended cell (0.2 mL) subcutaneously implanted into the left second breast fat pad of each mouse. Tumor growth was examined every 3 days, and tumor volume was calculated using the equation V = D × d^2^/2 (V, volume; D, long diameter; d, short diameter). When the average tumor size reached approximately 60 mm^3^, DDP was administered 2 times per week by intraperitoneal injection at a dose of 4.2 mg/kg. A month after the injection, mice were killed and the subcutaneous growth of each tumor was examined.

### Statistical analysis

The SPSS 19.0 software was used to perform the statistical analysis. *P* values less than 0.05 were considered significant. All the data were presented using “mean ± SD”, each experiment was repeated for three times [14].

## Results

### Drug resistance and the apoptosis of 231/DDP and 231/wt cell lines

**Fig. 1:**
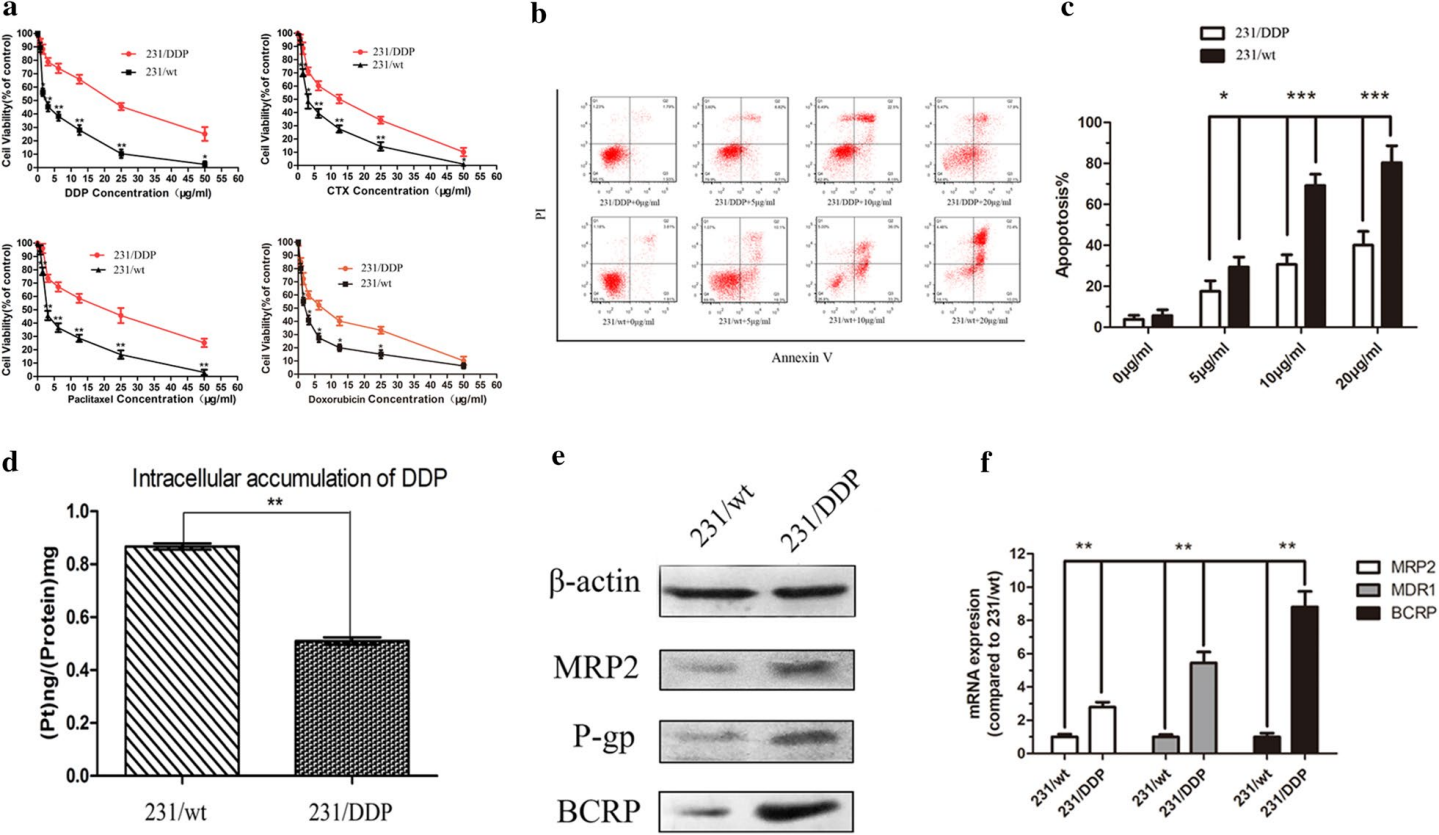
231/DDP cell is resistant to cisplatin. **a** MTT assay is used to evaluate the chemo-sensitivity of 231/wt cells and 231/DDP cells to different drugs of DDP/CTX/Paclitaxel/DOX after 48 h treatment (N = 3, **P < 0.001, *P < 0.01); **b**, **c** annexin V/PI assay is used to detect apoptosis in 231/wt and 231/DDP cell lines with different concentration of DDP; **d** difference on intracellular accumulation of DDP between 231/wt and 231/DDP cell lines. ICP-MS method is used to detect intracellular DDP accumulation in cells treated with DDP 1 μg/mL for 24 h, **P < 0.001. **e**, **f** ABC transporters protein expressions of MRP2, P-gP and BCRP in both of 231/wt cells and 231/DDP cells

To verify whether 231/DDP [8] had the resistant properties to DDP and multidrug, MTT method was used to detect cell inhibitory rate, namely IC_50_ of the aforementioned drugs (DDP, DOX, paclitaxel and cyclophosphamide). As presented in Fig. 1a, after 48 h treatment with DDP, the IC_50_ of the four drugs in 231/DDP cells were dramatically increased compared with MB-MDA-231 cells, especially the IC_50_ of DDP (Table 2). Annexin V/PI assay indicated that after 48 h cultivation with different concentration of DDP, the apoptosis ratio was significantly lower in 231/DDP group, than 231/wt group (Fig. 1b, c*, P *< 0.05).

**Table 2:** IC_50_ and RI of MDA-MB-231 cells and MDA-MB-231/DDP cells to different drugs of DDP/CTX/Paclitaxel/DOX after 48 h treatment

Group	IC_50_ value (μg/mL mean ± SD)			
	MDA-MB-231	MDA-MB-231/DDP	RI	*P*
CTX	3.97 ± 0.11	10.47 ± 0.42	2.64	< 0.001
Doxorubicin	2.38 ± 0.09	6.55 ± 0.41	2.75	< 0.001
DDP	3.13 ± 0.12	19.44 ± 0.89	6.21	< 0.001
Paclitaxel	4.08 ± 1.36	16.94 ± 1.69	4.15	< 0.001

### Intracellular accumulation of DDP and ABC transporters alterations in 231/DDP cell lines

Based on ICP-MS detection, we found intracellular DDP concentration was 0.51 ng/mg in the 231/DDP cell line after 24 h cultivation with 1 μg/mL DDP complete medium, significantly lower than that in the 231/wt cells (0.87 ng/mg, *P *< 0.01, Fig. 1d). To further verify the drug resistance, western blot and qRT-PCR was conducted to detect the ABC transporters expressions of MRP2, P-gP and BCRP, three known proteins related to drug resistance on membrane, in both of 231/wt cells and 231/DDP cells. As expected, mRNA and protein expressions of them were obviously increased in 231/DDP cells, compared with 231/wt cells (Fig. 1e, f).

### ETS1 was over expressed in 231/DDP cell line by microarray experiment, PPI network analysis and qRT-PCR

**Fig. 2:**
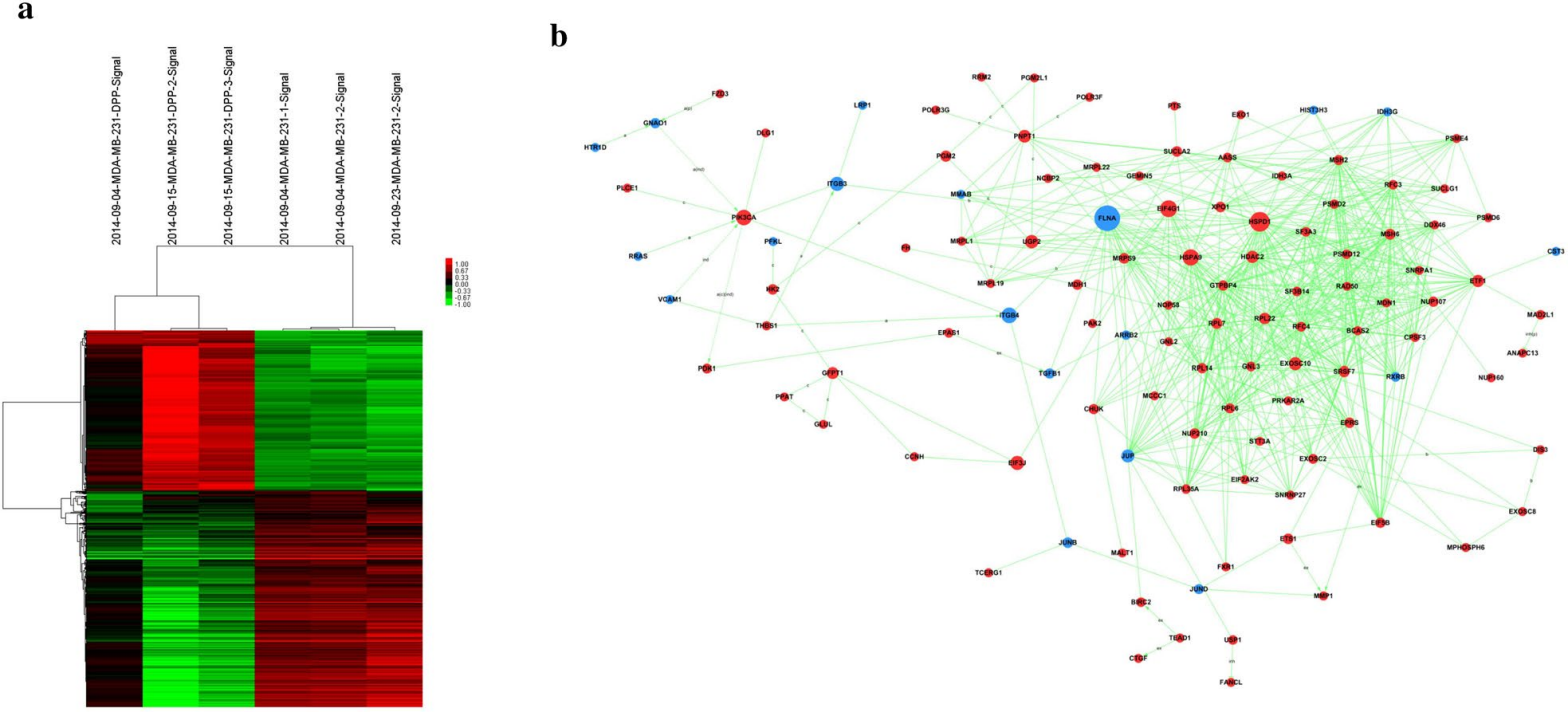
Microarray analysis and PPI network analysis. **a** DEGs between 231/wt and 231/DDP cell line using microarray analysis; **b** PPI network of the DEGs combining information in the STRING database

Based on the aforementioned selection criteria (P ≤ 0.05 and FC > 1), a set of 895 DEGs were identified between 231/wt cell line and 231/DDP cell line, which could well distinguish the two kinds of cell lines (Fig. 2a). Based on information in the STRING database, a PPI network for the DEGs was constructed. A node in the PPI network represents a protein product of a DEG, and an edge denotes an interaction between two genes. Degree of a node represents the interplayed protein numbers with this gene. Among the PPI network, several nodes had relatively high degrees, such as RPL6 (degree = 2), RBBP8 (degree = 3), ETS1 (degree = 7), BIRC2 (degree = 5), PIK3A (degree = 4) and RARS (degree = 4) (Fig. 2b). Integrating information in the PPI network with DEGs, the highlighted gene was used as a key word to search drug-resistant genes in databases such as the PubMed, in combination with words of “multidrug resistance (MDR)” or “drug resistance” OR “chemotherapy resistance”. The above six predominant nodes in PPI network have been reported in the literature, thus their expressions were validated in vitro.

**Fig. 3:**
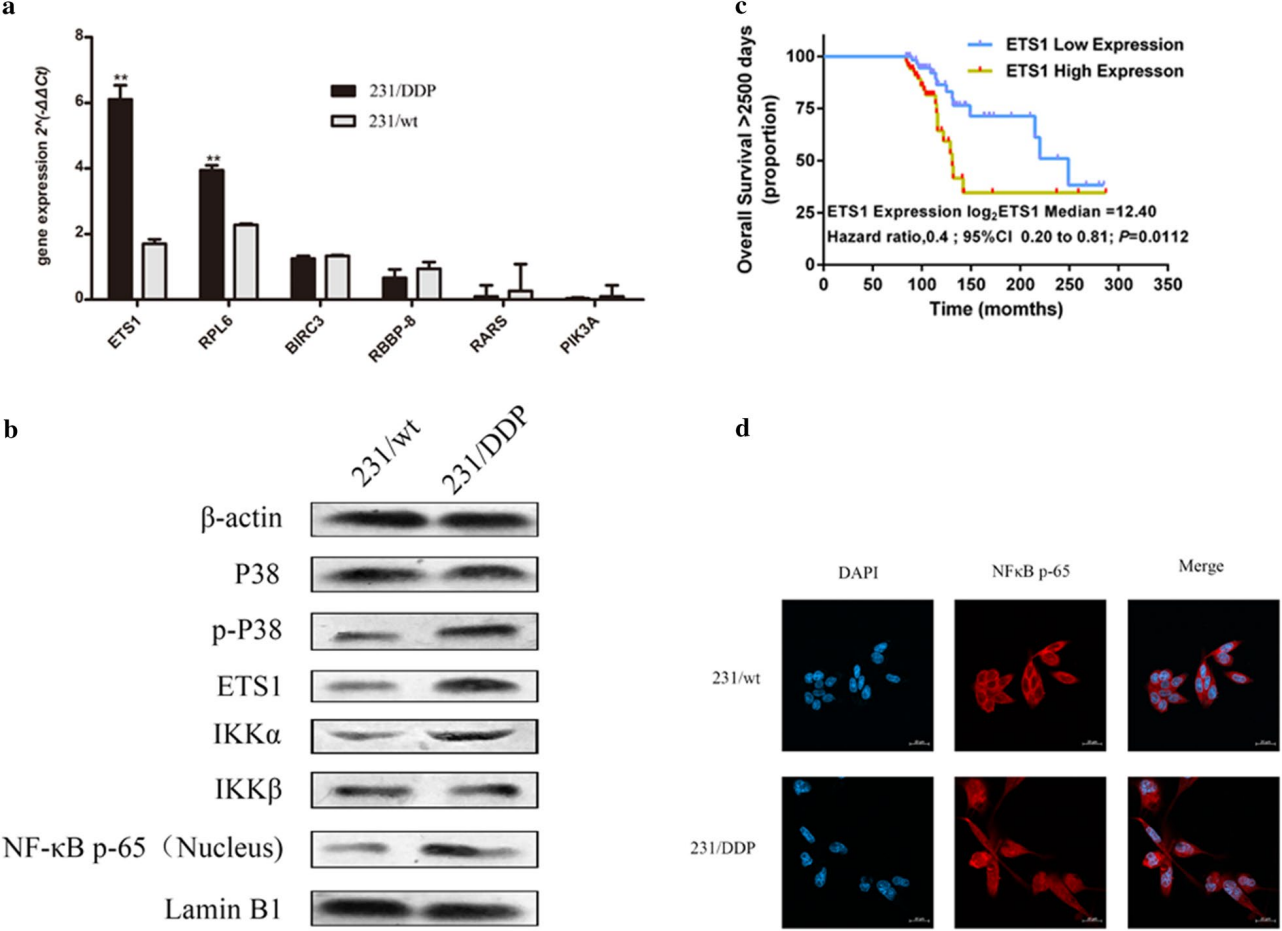
DDP drug resistance protein, activation of NF-κB pathway ETS1 mRNA expression is correlated with overall survival. **a** mRNA expression validation of six predicted drug-resistant genes via qRT-PCR; **b** 231/wt and 231/DDP cells were cultured in 6-well plates for 48 h. Western blot analysis was performed to measure the protein expression of P38, p-P38, ETS1, IKKα,IKKβ and NF-κB p-65 (nucleus) between 231/wt and 231/DDP cells. **c** Kaplan–Meier curve shows overall survival differences between relative low-expression ETS1 group and high-expression group (*P* = 0.0112). **d** Immmunofluorescence staining localization of NF-κB by antibody and nuclei staining by DAPI between 231/wt and 231/DDP cells

Results of qRT-PCR revealed that gene expressions of *ETS1*, *RPL6*, *BIRC3*, *RBBP8*, *RARS* and *PIK3A* in 231/DDP cells were all increased compared to 231/wt cells, especially the expression of ETS1 (Fig. 3a). Protein expression via western blot indicated that ETS1 protein was dramatically increased in 231/DDP cell line, compared with 231/wt cell line (Fig. 3b), suggesting this gene’s alteration at both mRNA and protein level was tied up with drug resistance to DDP in 231/DDP cell line.

### ETS1 expression was correlated with survival of BC

K–M curve [15] showed that the median survival of breast cancer patients in ETS1 low-expression group was 8.3 months, significantly longer than that in high-expression group with 4.4 months (*P* = 0.0112, Fig. 3c). Besides, the HR of the ETS1 level conducted by univariate Cox proportional hazards regression method was 0.401 (CI 0.198, 0.812).

### Cisplatin resistance was induced byETS1/IKKα/NF-κB signalling

The downstream effector of P38/ETS1 signaling was identified by cisplatin resistanc. The translocation of NF-κB in 231/DDP cells was determined by using immunofluorescence staining (Fig. 3d). In addition, the expression of P38, phosphor-P38, ETS1, IκB kinase α (IKKα) and IκB kinase β (IKKβ) as well as NF-κB was determined. Cisplatin resistance induced the high expression of ETS1, IKKα, NF-κB and phosphor-P38 (Fig. 3b). Moreover, it is noting that cisplatin resistance by the high expression of IKKα and further affecting up-regulation expression of NF-κB, not IKKβ which IKKβ is considered as classic path way to activate NF-κB [16].

### Inhibition of ETS1 expression enhances chemo-sensitivity to DDP in 231/DDP cell lines

**Fig. 4:**
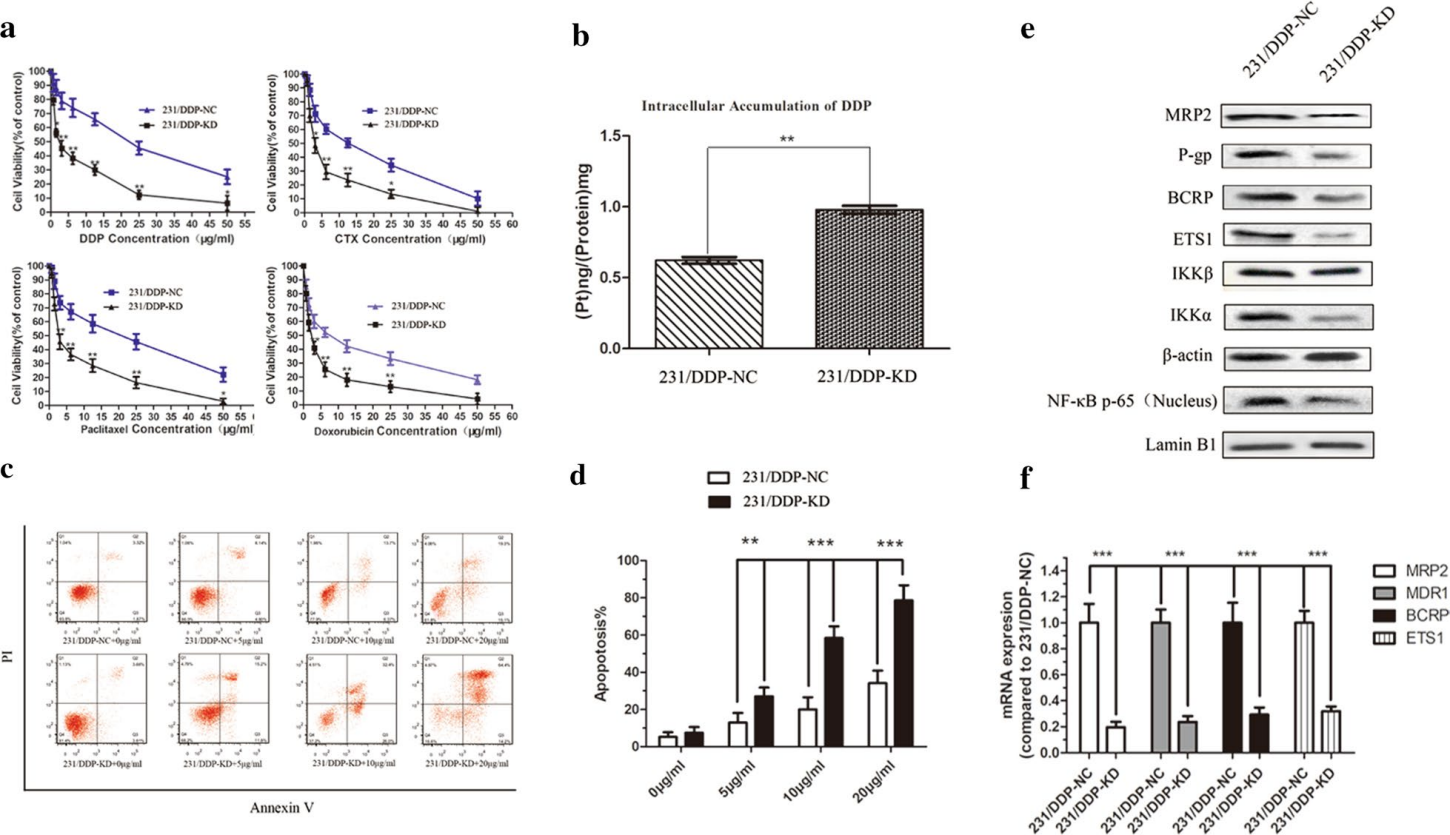
After ETS1 knocking down in 231/DDP cell line. **a** MTT assay is used to evaluate the chemo-sensitivity of 231/wt cell line and 231/DDP cell line transfected by shETS1 to different drugs of DDP/CTX/Paclitaxel/DOX after 48 h treatment (N = 3, **P < 0.001, *P < 0.01); **b** difference on intracellular accumulation of DDP in 231/wt and 231/DDP cell lines after siETS1 transfection. ICP-MS method was used to detect intracellular DDP accumulation in cells treated with DDP 1 μg/mL for 24 h, **P < 0.001; **c**, **d** annexin V/PI assay is used to detect apoptosis in 231/DDP-NC and 231/DDP-KD cell lines with different concentration of DDP; **e** proteins expression after ETS1 knocking down

After ETS1 knocking down, the resistance to four drugs as DDP, DOX, paclitaxel and cyclophosphamide in 231/DDP cell was remarkably reduced; especially to DDP, whose IC_50_ was significantly reduced from 19.62 to 2.92 (*P *< 0.001, Fig. 4a; Table 3). ICP-MS detection results showed that intracellular DDP concentration was significantly increased in 231/DDP-KD cell line, compared to that in the 231/DDP cell (*P *< 0.001, Fig. 4b). With different concentration of DDP, the apoptosis ratio was significantly higher in 231/DDP-KD cell lines than 231/DDP cell lines (Fig. 4c, d). These results suggest ETS1 inhibition enhances chemo-sensitivity to DDP in 231/DDP cell lines.

**Table 3:** IC_50_ and RI of MDA-MB-231/DDP, NC and ETS1-KD cells to different drugs of DDP/CTX/Paclitaxel/DOX after 48 h treatment

Group	IC_50_ value (μg/mL mean ± SD)			
	NC	ETS1-KD	RI	*P*
CTX	10.32 ± 1.63	3.47 ± 0.55	3.02	< 0.001
Doxorubicin	7.14 ± 1.08	2.42 ± 0.33	2.55	< 0.001
DDP	19.11 ± 3.65	2.92 ± 0.53	6.43	< 0.001
Paclitaxel	16.05 ± 3.16	3.89 ± 0.72	3.97	< 0.001

### Proteins expression after ETS1 knocking down

After ETS1 knocking down, protein expression of ETS1 was remarkably reduced in the 231/DDP cell line, compared to the blank control (NC) (Fig. 4e), indicating ETS1 expression was successfully inhibited. ABC transporters and IKKα as well as NF-κB were also decreased in 231/DDP cell with ETS1-KD (Fig. 4e), suggesting the 231/DDP cell line might be more sensitive to ETS1 alterations and ETS1 could be the upstream effector of IKKα and NF-κB.

### IKKα promoter activity and mRNA expression were regulated by ETS1 binding

**Fig. 5:**
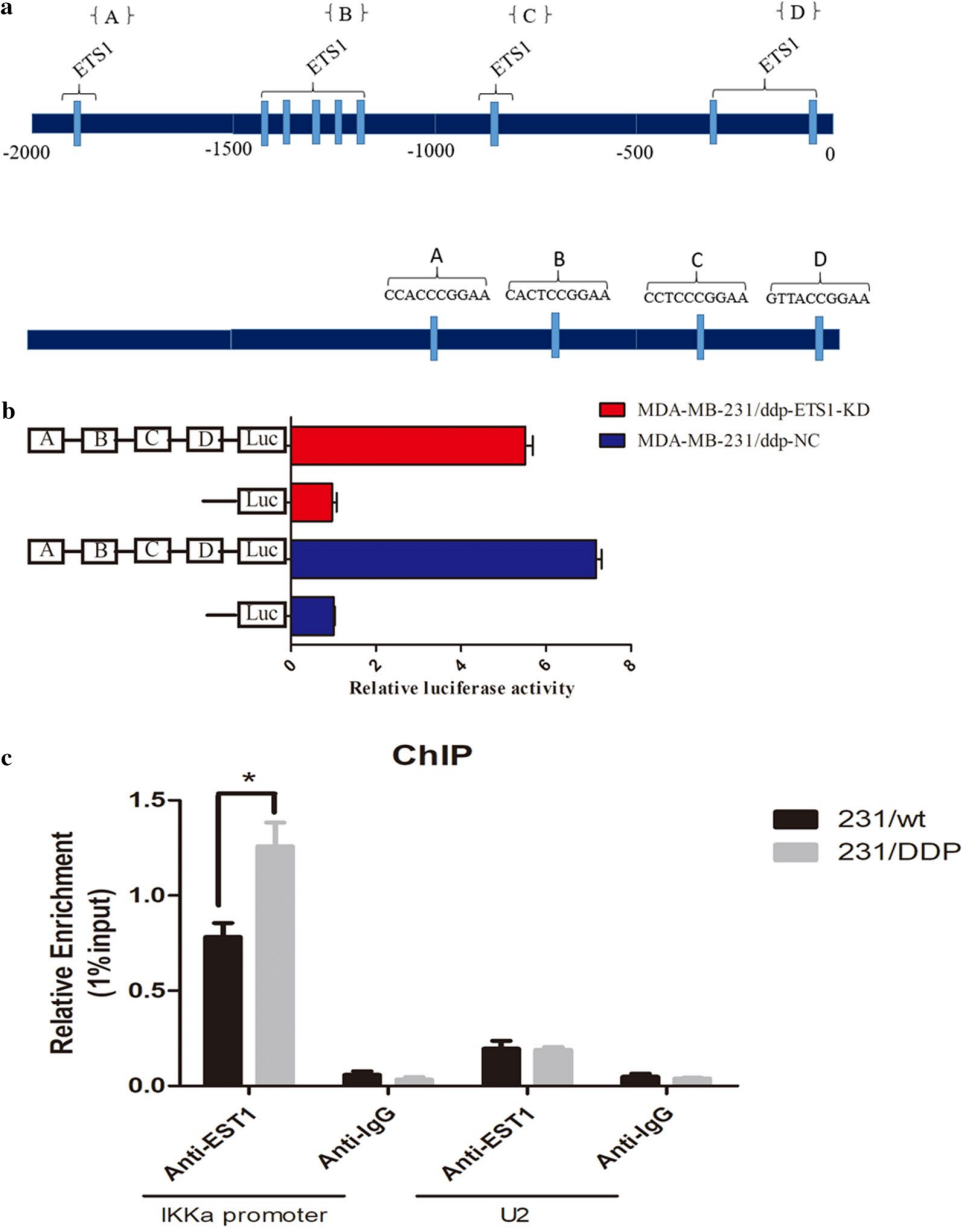
IKKα promoter activity and mRNA expression were regulated by ETS1 binding. **a** Indicated the presence of ETS1 transcription factor putative-binding sites that cluster into four main regions; **b** luciferase reporter assay revealed that IKKα-Luc in 231/DDP-NC was higher than 231/DDP-KD. **c** ChIP was performed using an anti-ETS1 antibody or a negative control IgG antibody in 231/DDP and 231/wt cells. Immunoprecipitated DNA was subjected to RT-qPCR using specific IKKα primers that included the ETS1 binding sites

Promoter analysis of IKKα, using PROMO TOOL V. 8.3 of TRANSFAC [17] (Beverly, MA, USA), indicated the presence of ETS1 transcription factor putative-binding sites that cluster into four main regions (Fig. 5a). To validate the elements for regulation in the IKKα promoter region by ETS1, we generated one pCDNA3.1-based luciferases constructs of IKKα containing clusters A, B, C, D (Fig. 5b). A luciferase reporter assay revealed that IKKα-Luc in 231/DDP-NC was higher than 231/DDP-KD. This results suggest that ETS1 has one or some clusters for regulatory promoter element for regulation of IKKα. ChIP assay was performed, In comparison with control IgG, the IKKα promoter had increased (3.3- to 7.8-fold) expression in 231/wt and 231/DDP. The result strongly induced ETS1 to bind to the IKKα promoter over time. No enrichment was observed in the control IgG group (Fig. 5c).

**Fig. 6:**
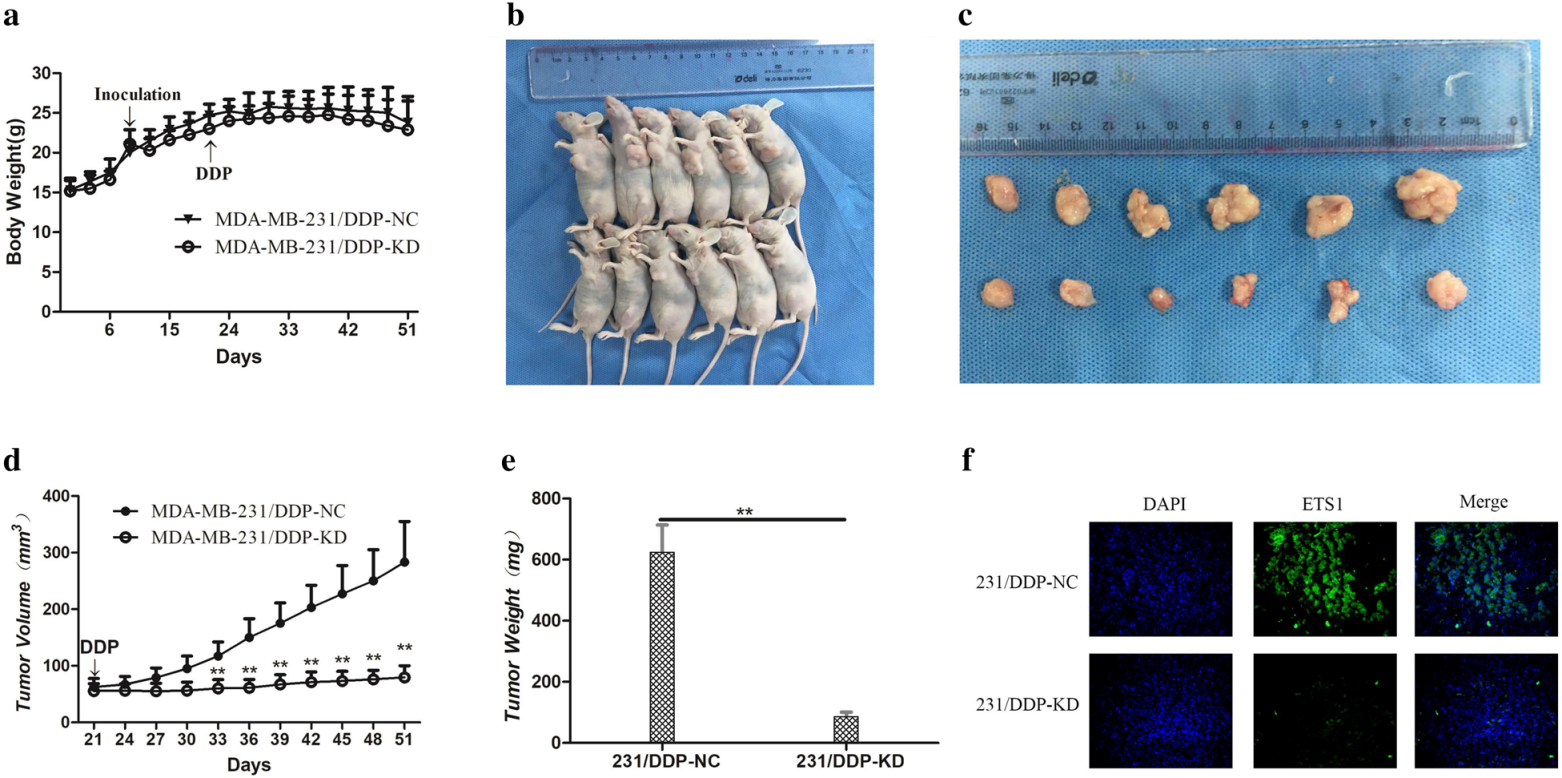
Down-regulation ETS1 improves the vivo sensitivity to DDP. **a** The average weight of mice were recorded every 3 days; **b**, **c** a month after initial cisplatin administration, the tumor volume and average weight receiving pCDNA-ETS1- or empty vector-transfected 231/DDP cells were recorded; **d** tumor volume was calculated every 3 days after cisplatin treatment; **e** a month after initial cisplatin administration, the tumor average weight receiving pCDNA-ETS1- or empty vector-transfected 231/DDP cells were recorded; *P < 0.05, **P < 0.01. **f** Tumors developed from PcDNA-ETS-transfected 231/DDP cells showed lower ETS1 protein levels by immunofluorescence staining; × 400

### Down-regulation ETS1 improves the vivo sensitivity to DDP

To further investigate the underlying roles of ETS1 in enhancing the chemosensitivity of TNBC cell to DDP, we used nude mice xenograft model. 231/DDP cells transfected with shETS1 or empty vector were subcutaneously injected into mice, and followed by treatment with DDP. A month after the initial DDP treatment, the body weight, volume and average weight of tumor xenograft was recorded (Fig. 6a–e). Immunofluorescence staining was used to detect localization and expression level of ETS1 in tumors tissue (Fig. 6f). As shown in Fig. 6d, the tumors formed from shETS1-transfected 231/DDP cells grew significantly more slowly than those from empty vector following DDP treatment. It was also observed that the down-regulation of ETS1 led to the inhibition of tumor growth. These results suggested that ETS1 low expression increased the in vivo chemosensitivity of TNBC cells to DDP.

## Discussion

Drug resistance is a major barrier in cancer treatment [18]. Although DDP has been applied for TNBC treatment, drug resistance is detected. In the present study, 231/DDP cell line had a higher IC_50_ value of DDP than 231/wt cell line (Fig. 1a). Meanwhile, 231/DDP cell line had a lower intracellular DDP concentration compared with 231/wt cell line, indicating 231/DDPcell line had a higher excretive ability of DDP, which might explain its strong drug resistance. Moreover, expressions of three drug-resistant membrane proteins (MRP1, P-gP and BCRP) [19, 20] were remarkably increased in 231/DDP cell line. Collectively, these might be the physiochemical and molecular basis of DDP-drug resistance mechanisms.

ETS1 is a TF regulates genes involved in stem cell development, tumor genesis and metastasis [21]. In drug-resistant MCF-7 breast cancer cells, ETS1 is overexpressed and results in up-regulation of MDR1 [22]. Thus, down-regulation of ETS1 via shRNA stably transfection might be a promising treatment for MDR breast cancer therapy [23]. MiR-200c counteracts trastuzumab resistance by suppressing TGF-β signaling and targeting ZEB1 in breast cancer [24]. ETS1 could regulate expression of ZEB1 [25], implying that targeting ETS1 might also counteract the drug resistance. In MCF-7/ADR cells, silencing of ETS1 by down-regulating MDR1 reverses adriamycin resistance [22]. Consistent with these previous studies, in our study, ETS1 was overexpressed in 231/DDP-resistant cell line via microarray analysis and expression validation experiment (Fig. 4). Meanwhile, down-regulation of ETS1 via shRNA stably transfection remarkably increased DDP response by reducing IC_50_ to DDP and increasing intracellular DDP concentration (Fig. 4). These give potent evidence that knockdown ETS1 might be an effective strategy to reverse DDP-resistance in TNBC.

In our study, we used public data deposited in TCGA database [26] and performed survival analysis in breast cancer with different ETS1 expressions, and found high ETS1 expression was significantly associated with poor survival in breast cancer (Fig. 3c), which was consistent with previous findings.

Activation of the NF-κB canonical pathway, leading to the translocation of NF-κB into the nucleus, is one of the strategies which leading cancer cells become drug resistance [27]. Signaling of ETS1/P38 has been proposed to play an important role in the development of tumor [25, 28–31]. Several studies have revealed that ETS1 is over-expression in drug resistant cancer cells [21–23]. As our research found that the high expression of ETS1 and IKKα, not IKKβ,in 231/DDP cells. On the other hand, the binding of ETS1 leads the down-regulation expression of IKKα and ABC transporters. Thus, we hypothesize that high expression of ETS1 may influence the activation of NF-κB [32], though the IKKα pathway not IKKβ and further leading to down-regulation of ABC transporters. Our team has proved it though luciferase assay and chromatin immunoprecipitation technique (ChIP).

## Conclusions

**Fig. 7:**
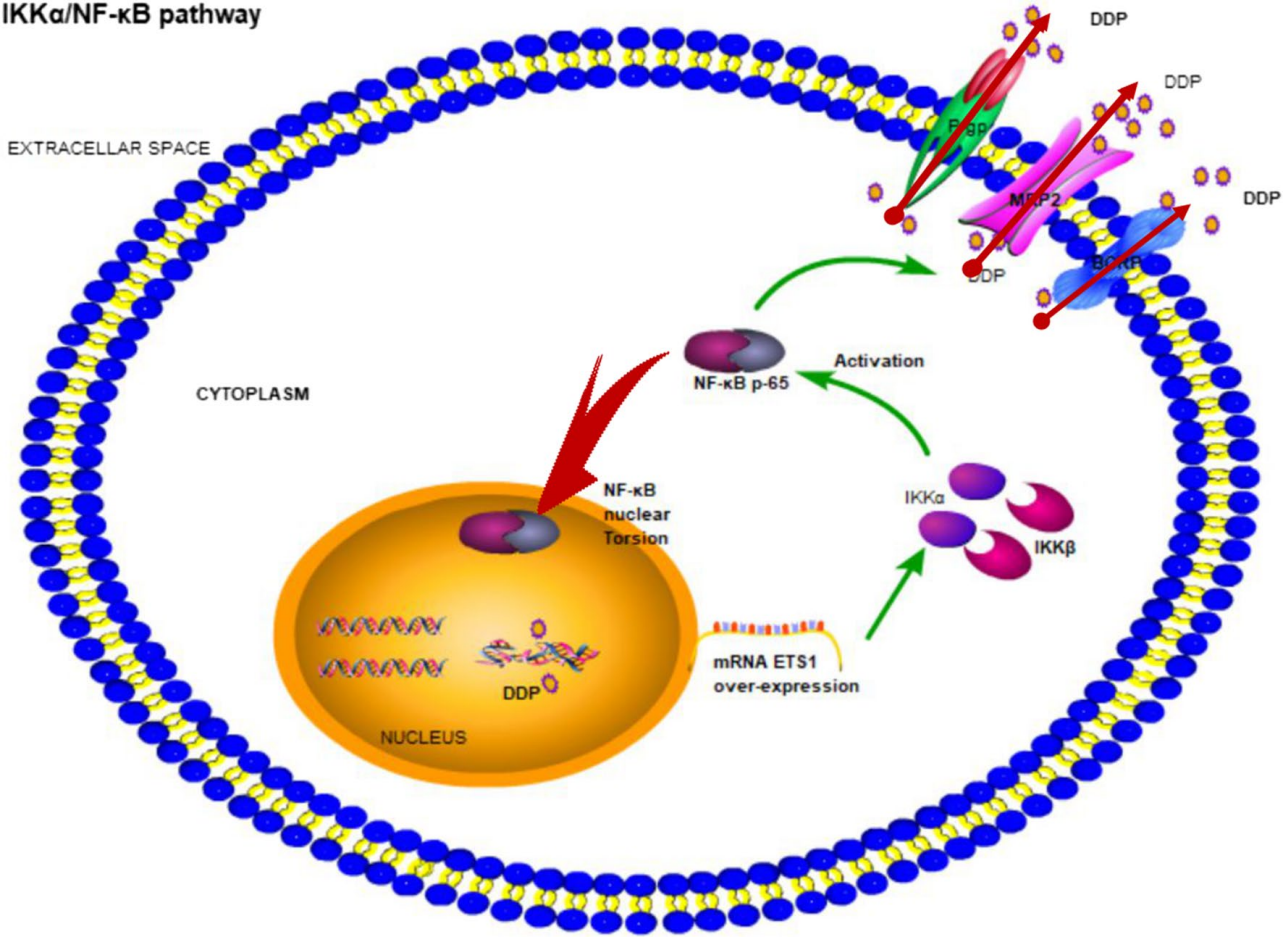
Proposed model for ETS1 activation of IKKα/NF-κB pathway inducing DDP resistance. Up-regulation of ETS1 activates IKKα, though its promoter sites binding, then activation NF-κB pathway which is a non-classic pathway. Next, the activation of NF-κB occurs nuclear translocation and increases expression of ATP transports which could be causing DDP resistance

Our data demonstrated that the dysregulation of ETS1 underlies the DDP resistance of TNBC, indicating that its overexpression may contribute to increased DDP chemosensitivity by enhancing ABC transporters via IKKα/NF-κB path way (Fig. 7). This study enables us to understand the functions of ETS1 in TNBC chemotherapy and suggests that ETS1 could be used as a novel marker of poor response to DDP and a potential therapeutic target for TNBC chemotherapy.

## Abbreviations

TNBC: triple-negative breast cancer
DDP: cisplatin ICP-MS:inductively coupled plasma mass spectrometry
ChIP: chromatin immunoprecipitation technique
231/DDP: MDA-MB-231/DDP
231/wt: MDA-MB-231
IC: inhibitory concentration
DOX: doxorubicin
CTX: cyclophosphamide
DMF: amide,n,n-dimethyl-formicaci
PBS: phosphate buffer solution
DMSO: dimethyl sulfoxide
qRT-PCR: quantitative real-time reverse transcription-PCR
